# Temperature directly and indirectly influences food web structure

**DOI:** 10.1038/s41598-019-41783-0

**Published:** 2019-03-29

**Authors:** Jean P. Gibert

**Affiliations:** Department of Biology, Duke University, Durham, NC 27708 USA

## Abstract

Understanding whether and how environmental conditions may impact food web structure at a global scale is central to our ability to predict how food webs will respond to climate change. However, such an understanding is nascent. Using the best resolved available food webs to date, I address whether latitude, temperature, or both, explain the number of species and feeding interactions, the proportion of basal and top species, as well as the degree of omnivory, connectance and the number of trophic levels across food webs. I found that temperature is a more parsimonious predictor of food web structure than latitude. Temperature directly reduces the number of species, the proportion of basal species and the number of interactions while it indirectly increases omnivory levels, connectance and trophic level through its direct effects on the fraction and number of basal species. While direct impacts of temperature are routinely taken into account to predict how ecosystems may respond to global climate change, indirect effects have been largely overlooked. These results thus suggest that food webs may be affected by a combination of biotic and abiotic conditions, both directly and indirectly, in a changing world.

## Introduction

Food web structure can affect the dynamics and stability of large species assemblages (e.g.^1–3^) as well as the flow of energy and matter across ecosystems (e.g.^4,5^). Because of that, understanding the factors that determine such structure is a central and long-standing goal of ecology^1,3,6–8^. Multiple biotic factors are known to influence food web structure, like body size and allometric scalings^9–12^, genetic and phenotypic variation^13,14^, and the number and nature of predator prey interactions^2,15–17^. While environmental conditions are known to influence the building blocks of food webs –predator-prey interactions and dynamics^18–24^– how abiotic factors across latitudinal gradients may broadly influence food web structure is still poorly understood^25,26^, but a pressing issue in times of rapid global climate change.

Temperature is one important factor known to change with latitude. Some theoretical studies suggest that temperature impacts on food web structure can be difficult to predict due to the potential for idiosyncratic temperature responses of the species embedded within food webs^27^. Other studies suggest the possibility of specific impacts, such as a decrease in the number of trophic levels due to changes in underlying controlling phenotypic traits^25^ as well as changes in food web connectance, due to temperature impacts on feeding interactions^28^. But latitude also has the potential to influence food web structure independently of temperature. Seminal work by Elton^29^ suggested that the number of trophic levels could be controlled by energetic subsidies, such that more productive environments at lower latitudes would have longer food chains than less productive environments at higher latitudes^30^ (or the flipside, that larger ecosystems could harbor longer food chains than smaller ones)^31,32^. Other effects of latitude are possible, as species richness is well known to change with latitude, and there is potential for broad biogeographic patterns to play a role into which species are present in food webs across latitudinal gradients. Latitudinal differences in niche breadth among temperate and tropical species could also lead to differences in the number of interactions per species^33,34^, food web connectance (a measure of how interconnected the network is), and the number of trophic levels. Because of the potential for independent effects of temperature and latitude, but also because latitude is a major determinant of annual mean temperatures, it is increasingly important to assess how both factors interplay to influence food web structure.

Empirical studies linking changes in latitude and temperature to food web biomass structure across trophic levels have so far led to conflicting results. Early experimental work showed that top predators and intermediate species are more susceptible to changes in temperature than primary producers, which results in warmer food webs being species-poor and bottom-heavy –or “greener”^35^. More recent work, however, suggests that differential responses to temperature by consumers and producers may lead to increased levels of top-down control, and thus, lower, not larger, primary producer biomass^36,37^. But while increases in top-down control with temperature towards the tropics were also observed in marine food webs^19^, top-down control has been shown to increase, not decrease, the biomass of basal species in warmed coastal food webs^38^. Moreover, temperature has also been shown to weaken, not strengthen, top-down control in tundra soil food webs dominated by spiders and collembolans, which led to biomass accumulation at lower trophic levels^39^.

Global analyses of changes in food web network structure with latitude, temperature and ecosystem type, have also led to conflicting results. Food chain length in aquatic systems was shown to only very mildly vary with latitude, if at all^30^, while a large scale meta-analysis suggests that ecosystem type, but not latitude, impacts food web structure^34^. On the other hand, systematic sampling of pitcher plant food webs across a continental-scale latitudinal gradient showed that both the number of species and the number of interactions per species increased with latitude. Despite these results, low amounts of total explained variation led the authors to conclude that food web structure was broadly independent of abiotic climatic factors^40^. Together, these results suggest that not only do we still lack a general understanding of how latitude and temperature influence food web structure, but we also have so far not been able to tease apart their potentially independent effects from their joint effects.

Here, I aim to address these issues by analyzing a compilation of some of the best resolved food webs to date from across the globe. In particular, given the lack of support in the literature for potential latitudinal niche-breadth effects^30,34^, I test whether variation in food web structure is more strongly correlated with temperature, latitude, both or none. While my results suggest that temperature and latitude can both have direct and indirect effects on different aspects of food web structure, a model only taking temperature into consideration is more parsimonious than one considering both latitude and temperature. In light of this, I tested a few additional hypotheses.

First, while warming may lead to the loss of top predators and an increase in the proportion of basal species^35,41,42^, temperature is also known to increase grazing and top-down control through physiological effects (e.g.^21,24,43,44^), which can in turn decrease the standing biomass of primary producers^37^ and the proportion of basal species^36^. Because of this potential for antagonistic effects, I tested the following two alternative hypotheses: either an increase in temperature leads to a larger fraction of basal species that is consequence of food web simplification and release from predation, or it leads to increased top down control, which in turn reduces the number and fraction of basal species. Second, a smaller (larger) fraction of basal species means a larger (smaller) fraction of top and intermediate species, hence, more (less) consumers and more (less) predator-prey interactions. I thus tested the following two alternative hypotheses: either increasing temperatures may indirectly decrease the total number of interactions through increasing the fraction of basal species, or it may indirectly increase those interactions through decreasing the fraction of basal species. Third, and as a consequence of the previous hypotheses, I tested whether as temperature indirectly decreases (increases) the number of interactions through its impact on the fraction of basal species, it may also decrease (increase) the degree of omnivory and food web connectance (a measure of how densely connected the network is), as both these metrics typically increase with a larger number of feeding interactions. Because more interconnected food webs also have a larger number of trophic levels (e.g.^45^), a decrease (increase) in omnivory and connectance due to indirect temperature effects should also be accompanied by a decrease (increase) in the number of trophic levels.

## Methods

### The data

**Figure 1:**
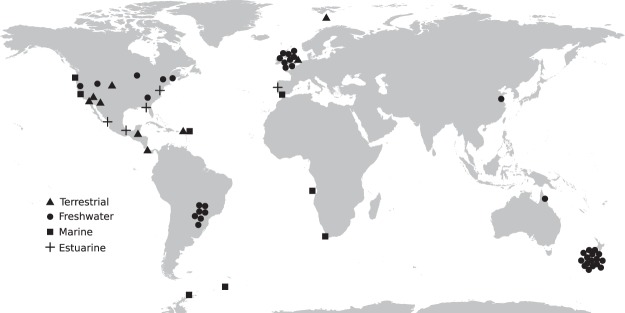
Map of the locations of all used food webs by ecosystem type (Terrestrial, Freshwater, Marine, Estuarine). For Brazil, United Kingdom and New Zealand, only approximate locations are shown as to also illustrate the number and type of food webs considered in each loction.

Food webs were taken from the Interaction Web Database (https://www.nceas.ucsb.edu/interactionweb/), the GlobalWeb food web database (https://www.globalwebdb.com/), and the R package *cheddar*^46^. Because food webs typically vary greatly in their resolution, I only kept those with at least 25 species and 50 interactions. The final dataset comprises a total of 65 food webs, averaging 64 species and 427 interactions, including some of the best resolved in the world^14^ (Fig. 1, Table S1 in Appendix 1). For each food web, I obtained latitudes and longitudes from the original studies or estimated the coordinates based on the reported locations. Using those coordinates, I obtained global surface temperatures (annual average) from BioClim GIS layers (http://www.worldclim.org/bioclim, BIO1 layer)^47^, for terrestrial and aquatic ecosystems on continents, and, for marine food webs, I used ocean surface mean temperatures from the dataset *levitus*, in R package “ocedata”^48,49^, originally compiled from the 2013 version of the World Ocean Atlas. Temperatures were unavailable from GIS layers for 7 food webs (Antartica, Chesapeake, Monterey Bay, Stony Stream, Sutton Au, Sutton Sp, Sutton Su, Table S1). In those cases, I estimated annual average temperatures using data from other publicly available climate databases (details in Appendix 2). Excluding those 7 food webs did not alter the results (see Results section).

### Descriptors of food web structure

Food webs vary in their degree of taxonomic aggregation. That aggregation has been shown to bias some measures of food web structure like the fraction of top species, multiple measures of trophic chain length and the number of trophic levels, as well as the number of feeding interactions^50,51^. To control for the imperfect nature of food web data, I aggregated taxa into trophospecies –or sets of redundant taxa in terms of their structural role–, following previous studies (e.g.^50,52,53^). However, the impacts of temperature (and perhaps even latitude) can be species-specific^21^, and so, there is no reason to believe that trophospecies are in any way a meaningful level at which to measure, or even expect to detect, the effects of temperature. All analyses where thus performed in both non-aggregated and aggregated food webs.

I operationally defined the number of species (or trophospecies), the number of feeding interactions (called links hereafter), and the proportion of basal, intermediate and top species, as biotic measures of food web structure. The proportion of basal and intermediate species were strongly negatively correlated, so I only kept the proportion of basal and top species in all analyses (Appendix 3). In addition, I quantified the directed connectance of all food webs (Links/Species^2^), which measures the proportion of realized interactions, from all possible ones, including cannibalism. I also calculated the maximum trophic level using the standard relation for unweighted food webs,

$$max\,(T{L}_{i}=1+1/{n}_{i}\sum _{j=1}^{S}\,T{L}_{j})$$

where the focal trophic level (*TL*_*i*_), is a function of the trophic level of each consumed species (*TL*_*j*_), *S* is the number of species in the food web and *ni* is the total number of prey items for species *i*. This metric thus assumes that predators feed equally among all its prey (e.g.^53^). While the mean trophic level of the food web could also have been used, maximum and mean trophic levels were strongly correlated (Appendix 3), so I only kept the maximum trophic level for all final analyses. Last, I quantified the levels of omnivory present in each food web as the fraction of species feeding upon multiple trophic levels^52^. For simplicity, I call connectance, maximum trophic level, and omnivory levels, the network-structural aspects of food webs. This said, all three metrics are obviously consequence of the number of species and links, as well as the proportion of basal, intermediate and top predators, and, hence, are also biotic in nature. While other metrics of food web structure exist, they were not directly tied to the hypotheses tested in this paper and were not considered.

### Statistics

Abiotic factors such as latitude and temperature can impact biotic aspects of food web structure like the number of species, the number of links, as well as the proportion of basal or top species. These biotics factors can in turn influence network-structural aspects like connectance, omnivory levels or trophic level. These effects can be direct (variable on variable), or indirect (through another variable)^54^. Because of this, I used a statistical framework that allowed me to: (1) assess how these biotic and network-structural aspects of food web structure jointly responded to the abiotic explanatory variables (temperature, latitude), (2) partition the effects of latitude and temperature on food web structure, as well as explicitly account for the fact that latitude is an important determinant of global temperatures, and, (3) explicitly model the causal relationships among the many aspects of food web structure (species, links, basal and top species, omnivory, connectance and trophic level) as well as both their direct and indirect effects. To do so, I used a suitable multivariate approach, structural equation modeling (SEM), with latitude, temperature, both or none, as explanatory variables, and the number of species, links, the fraction of basal and top species, omnivory levels, connectance and trophic level, as response variables, as well as the possible causal effects among all considered biotic and network-structural properties of the food webs (see Table S2 in Appendix 3 for model equations). SEM modeling also allows to partition and tease apart the direct, indirect (through another variable), and total effects (sum of direct and indirect) of the different variables (see Appendix 3 for details on how to calculate indirect and total effects).

When latitude and temperature were considered together in the same model, I explicitly accounted for latitude influencing annual average temperature at a global scale. Additionally, I included the effect of ecosystem type (aquatic or terrestrial) as another abiotic explanatory variable for both biotic and network-structural aspects of food webs (Table S2). While I acknowledge that food webs can occur in more than just two possible ecosystem types, the best and most widely used R package currently available for SEM modeling, *lavaan*^55^, can only account for continuous or binary variables. Because the different variables all had different magnitudes and variance, they were all standardized to Gaussian distributions of mean equal to one and zero variance by subtracting the sample mean to each data point and dividing by the sample standard deviation. SEM modeling was done with package *lavaan* in R v3.5.0^48^. All data and code can be found in https://github.com/JPGibert/Temp_food_webs.

## Results

While all models fitted the data very well (Table 1), the model that only included temperature as an abiotic correlate was the most parsimonious (Table 1). However, the one including both latitude and temperature in all cases explained the most variance (Table 1, Appendices 4 and 5). These results were largely consistent for both aggregate and non-aggregate food webs (Appendix 4) and taking or not into account the 7 food webs for which temperature was not available from GIS layers did not alter the results presented here (Appendix 5).

**Table 1:** Structural equation model and model descriptors by model (with both latitude and temperature, only latitude or temperature, or neither) ranked by model delta AIC score.

SEM Model	*χ* ^2^	df	p-val	Comparative Fit Index	Root Mean Square Error of Approximation	Standardized Root Mean Square Residual	Adjusted Goodness of fit	AIC	ΔAIC
Temperature	1.804	3	0.614	1	0.000	0.016	0.894	906	0
Latitude	1.882	3	0.597	1	0.000	0.016	0.884	914	7
None	2.328	3	0.507	1	0.000	0.019	0.877	932	26
Temp + Lat	1.896	4	0.755	1	0.000	0.018	0.989	963	57

I report model chi-square values (*χ*^2^), degrees of freedom (df), p values (here, the larger the better), comparative fit square values (the closer to 1 the better), root mean square errors of approximation (the closer to 0, the better, values above 0.08 are suggestive of a bad fit), standardized root mean square residuals (smaller than 0.08 suggest a good fit), adjusted goodness of fit (can be interpreted as the proportion of explained variance), Aikaike Information Citerion values (AIC) and delta AICs (ΔAIC). For full model descriptions see Table S2 in Appendix.

**Figure 2:**
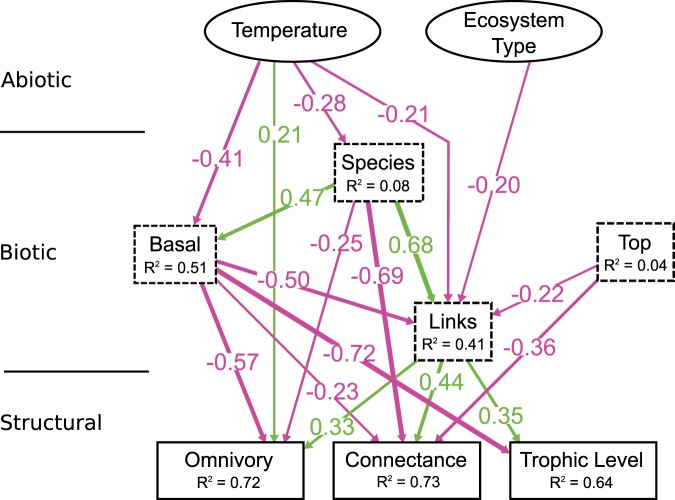
Standardized effects for the most parsimonious model (temperature only). For simplicity, only direct effects are shown, but indirect effects are depicted in Fig. 2. Abiotic factors (temperature, ecosystem type) depicted as solid ellipses, biotic factors (proportion of basal and top species, the number of species and links) as dashed rectangles, and measures of food web network structure (omnivory, connectance and maximum trophic level) as solid rectangles. Only significant effects are reported. Explained variance for each response variable is indicated as R^2^ values and all relevant statistics for these models can be found in Table 1. Pink arrows indicate negative effects while green arrows indicate positive effects.

**Figure 3:**
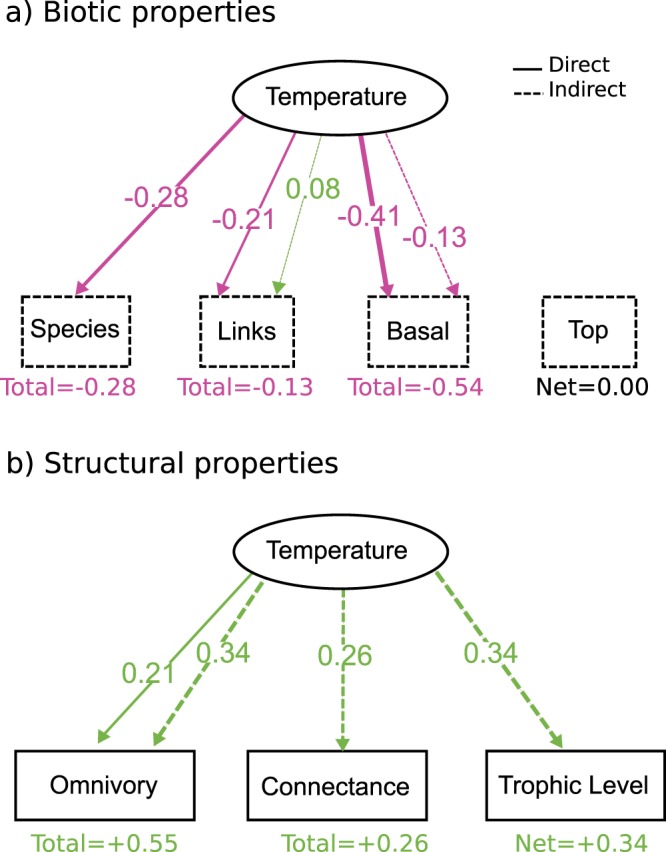
Direct and indirect effects of temperature on food web structure. Color coding as before. Solid lines represent direct effects while dashed lines represent indirect effects. All coefficients are standardized.

In the most parsimonious model (temperature only), temperature effects on food web biotic and network structural properties were many and various: first, temperature was directly correlated with a smaller total number of species, a smaller proportion of basal species, and a smaller number of links (Fig. 2). Second, among network-theoretical aspects of food web structure, temperature was directly correlated with larger omnivory levels (Fig. 2). Third, temperature was indirectly correlated with larger connectance and trophic level (Fig. 3) through its direct effects on the number of species, the proportion of basal species and the number of links, which were all directly and indirectly correlated to omnivory levels, connectance and trophic level (Fig. 2).

Interestingly, both the nature and the sign of temperature effects varied between biotic aspects of food web structure (species, fraction of basal species and links, Fig. 3a) and network-structural aspects (omnivory, connectance and trophic level, Fig. 3b). Indeed, temperature effects on biotic aspects were mostly direct and negative (Fig. 3a), while temperature effects on food web network-structural aspects were mostly indirect (through other variables) and positive (Fig. 3b), with the exception of omnivory which experienced both direct and indirect effects.

Ecosystem type only impacted the number of links (more links in aquatic food webs than in terrestrial ones) and no abiotic variable explained the proportion of top species, which was in turn negatively correlated with the number of links and connectance (Fig. 2). However, neither the effect of ecosystem type nor that of the top fraction of species was consistent among aggregated food webs or a dataset that did not consider the 7 food webs for which temperature was not available from GIS layers (Appendices 4 and 5).

## Discussion

Understanding how latitude and temperature directly and indirectly influence food web structure is an important and pressing goal of ecology in times of rapid, global climate change. Yet, this understanding is still nascent^19^. Previous studies have found no effect of latitude on food chain length^30^ or other food web structural patterns^56^, and because latitude is a good predictor of temperature, they inferred that temperature (and other climatic variables) had no effect on food chain length or other structural features. Others, however, found an increase in trophic level and overall connectivity with latitude, suggesting the potential for conflicting results^40^. My results add to this growing literature, showing that a model that only includes temperature as an abiotic explanatory variable of food web structure is more parsimonious than one that includes both latitude and temperature, only latitude, or none. However, a model with both latitude and temperature explains a larger fraction of the total variance, which implies that neither one fully explain food web structure, but the interplay between the two may.

### Temperature effects on food web structure

While previous studies have found both positive^36,37^ and negative^25,35^ impacts of temperature on basal species, my results support the hypothesis that temperature is directly correlated with a decrease in the proportion of basal species (Figs 2 and 3). A potential explanation of this pattern involves the larger metabolic costs associated with warmer temperatures, which results in stronger grazing and top-down control, as shown in previous studies^20,24,44,57^. However, the dataset used likely reflects thousands if not millions of years of species coevolution^58^ and local adaptation to their current climates. Because of this, it may not be advisable to use these results to predict short term responses to acute, rapid temperature shifts, but it is possible that these results may shed some light into possible long-term trends of food web structure after sustained periods of warming.

A second set of alternative hypotheses stated that a smaller (larger) fraction of basal species due to temperature would lead to a larger (smaller) total number of interactions. While the fraction of basal species was negatively correlated with the total number of interactions (Fig. 2), I found that temperature had only a marginally positive indirect effect on the number of links (through its effect on the total number of species and the fraction of basal species). Surprisingly, temperature had a larger direct negative effect on the number of links, which resulted in a net total negative effect (Fig. 2a). This result thus supports neither one of the original hypotheses, and highlights the importance of taking the multiple possible direct and indirect impacts of abiotic factors into account in order to fully understand their influence on food web structure. These results also suggest that in warmer food webs, predators have less interactions than in colder food webs, which is in accordance with the niche breadth hypothesis^33,34^: tropical species have narrower niche breadths, which leads to a larger number of specialists^59–61^ (with only one or a few interactions), while more temperate ecosystems should be dominated by generalist species, with wider niche breadths^59–61^ (broader diets, thus, more connections). These patterns of connectivity have nevertheless been seldom tested empirically^59^, so there is still more research needed on the topic.

The last set of alternative hypotheses involved how temperature would increase (decrease) omnivory levels, connectance and the number of trophic levels indirectly through increasing (decreasing) the number of links. Interestingly, temperature was directly associated to a lower, not a larger number of links (Fig. 2a), but had overall positive indirect effects on omnivory, connectance and the number of trophic levels, which supports, again, neither of my original hypotheses. While seemingly counterintuitive, there is a simple explanation for such a pattern when we consider both direct and indirect effects. First, the number of links directly increases connectance (as well as omnivory and trophic level, Fig. 2), but because temperature directly reduces the number of links, it would seem like it should indirectly reduce connectance, omnivory and trophic level. However, temperature also negatively influences the fraction of basal species and the total number of species, which both have strong negative effects on omnivory, connectance and trophic level (Fig. 2). This results in strong overall positive indirect effects of temperature on connectance, omnivory and trophic level (Fig. 3), which offsets the original expectation that was only based on the direct effect of temperature on the number of links. These data therefore suggest, once again, that temperature effects are rather complex, and that direct effects are as important as indirect effects to understand how these abiotic factors shape food web structure.

### Caveats

It is important to notice that while this dataset covers all continents (Fig. 1), food web data is currently unavailable in multiple areas of the globe (e.g., large portions of Asia and Africa only have so many food webs), and, as such, the global implications of these results need to be taken with caution. Second, there is a tremendous amount of variability in how different researchers and research teams compile food webs. Some of the resulting bias was taken care of using aggregated food webs (see Appendix 4) but some surely still persist. An alternative way to control for these biases would be to use mixed effects SEMs, with research group as a random variable, but the dataset is unfortunately too small to that end. Third, the temperature at the moment these food webs were compiled in the field may or may not match the annual average temperatures obtained from GIS layers. Moreover, temperatures fluctuate from year to year and can also do so seasonally. Temperature variability may thus be an important factor influencing food web structure but these analyses do not take that into account. Last, other environmental variables like precipitation or seasonality in precipitation may influence food web structure as well. As such, these results need to be considered as an important step towards understanding how temperature may influence food web structure, but more research is still needed.

## Conclusions

Overall, my results suggest that temperature can strongly influence food web structure through direct negative impacts on the number of species, the fraction of basal species and the number of feeding interactions, while still having indirect positive effects on omnivory levels, connectance and trophic level. Because temperature is known to have potentially antagonistic, asymmetric^62^ and species-specific effects^21^, my results suggest that we may need to consider its multiple direct and indirect effects to fully understand and predict food web responses to changes in environmental factors in a rapidly changing world.

## Supplementary information

Supplementary Information
