# Supplementary material for: Temperature directly and indirectly influences food web structure
Source: Sci Rep. 2019 Mar 29;9:5312. doi: 10.1038/s41598-019-41783-0 (PMC6441002; doi:10.1038/s41598-019-41783-0)
Supplement: Supplementary file 1 — Supplementary Information [file 41598_2019_41783_MOESM1_ESM.pdf]

Appendix: Temperature directly and indirectly influences food web structure

Jean P. Gibert[1],*
[1]Department of Biology, Duke University, Durham, NC 27708, USA
*jean.gibert@duke.edu

INDEX

1) Food web data 2-4

2) Temperature estimates when unavailable from GIS layers 5

3) Statistical Methods 6-9

4) Results for aggregated food webs 10-12

5) Results without the FWs for which Temp was unavailable from GIS 13-15

1) Food web data

Table S1: All food webs used, with some summary descriptors, the country of provenance, continent, ecosystem, type of food web and original reference.

FW name	Country	Continent	Ecosystem	Ecosystem Type	S	L	C	Ref
Afon Hafren	Wales/UK	Europe	Stream	Freshwater	25	135	0.216	Layer et al 2010
Aire	UK	Europe	Stream	Freshwater	60	185	0.051	Percival & Witehead 1929
Allt a'Mharcaidh	Scotland/UK	Europe	Stream	Freshwater	40	334	0.208	Layer et al 2010
Alvarado	Mexico	North America	Lagoon, Estuarine	Estuary	30	229	0.254	Cruz-Escalona et al 2007
Angola	Angola	Africa	Marine	Marine	28	127	0.161	Angelini & Vaz-Velho 2011
Antarctica	Chile/Argentina/UK claimed region	Antarctica	Marine	Marine	28	218	0.278	Cornejo-Donoso & Antezana 2008
Bear	Norway	Europe/Arctic	Island	Terrestrial	31	43	0.044	Hodkinson & Coulson 2004
Benguela	South Africa	Africa	Marine	Marine	29	203	0.241	Yodzis 1998
Bere Stream	England/UK	Europe	Stream	Freshwater	66	943	0.216	Layer et al 2010
Berwick	New Zealand	Oceania	Stream	Freshwater	77	240	0.040	Thompson & Townsend 2003
Black Rock Stream	New Zealand	Oceania	Stream	Freshwater	85	373	0.051	Townsend et al 1998
Braco Morto	Brazil	South America	River	Freshwater	39	248	0.163	Angelini et al 2013
Bridge Brook	US	North America	Lake	Freshwater	75	553	0.098	Havens 1992
Broad Stream	New Zealand	Oceania	Stream	Freshwater	94	565	0.063	Townsend et al 1998
Broadtsone	England/UK	Europe	Stream	Freshwater	25	178	0.284	Layer et al 2010
Cadiz	Spain	Europe	Marine	Marine	44	413	0.213	Torres et al 2013
Canton Creek	New Zealand	Oceania	Stream	Freshwater	108	708	0.060	Townsend et al 1998
Carpintería	US	North America	Marsh	Freshwater	128	2290	0.139	Lafferty et al 2006
Chesapeake	US	North America	Estuary	Estuary	33	72	0.066	Baird & Ulanowicz 1989
Coachella Valley	US	North America	Desert	Terrestrial	30	290	0.322	Polis 1991
Coral Reef	US/UK	Caribean/Antilles	Marine	Marine	50	556	0.222	Opitz 1996
Corrente	Brazil	South America	River	Freshwater	13	34	0.201	Angelini et al 2010
Coweeta 1 (Cooper)	US	North America	Stream	Freshwater	58	126	0.037	Thompson & Townsend 2003
Coweeta 17 (Herlzler)	US	North America	Stream	Freshwater	71	148	0.029	Thompson & Townsend 2003

Deep Creek	US	North America	Cool desert stream	Freshwater	32	140	0.136	Koslucher & Minshall 1973
Dempsters Autumn	New Zealand	Oceania	Stream	Freshwater	83	415	0.060	Thompson & Townsend 2000
Dempsters Spring	New Zealand	Oceania	Stream	Freshwater	93	538	0.062	Thompson & Townsend 2000
Dempsters Summer	New Zealand	Oceania	Stream	Freshwater	107	966	0.084	Thompson & Townsend 2000
Duddon Pike Beck	England/UK	Europe	Stream	Freshwater	35	286	0.233	Layer et al 2010
Hardknott Gill	England/UK	Europe	Stream	Freshwater	44	386	0.199	Layer et al 2010
Healy Creek	New Zealand	Oceania	Stream	Freshwater	96	634	0.068	Thompson & Townsend 2000
Huizache	Mexico	North America	Lagoon, Estuarine	Estuary	26	189	0.279	Zetina-Rejon et al 2003
Itaipu (83-87)	Brazil	South America	Freshwater reservoir	Freshwater	32	139	0.135	Angelini et al 2006
Itaipu (88-92)	Brazil	South America	Freshwater reservoir	Freshwater	33	141	0.129	Angelini et al 2006
Kye Burn	New Zealand	Oceania	Stream	Freshwater	98	629	0.065	Thompson & Townsend 2003
Las Cuevas	Belize	Central America	Deciduous Seasonal Forest	Terrestrial	165	114	0.004	Lewis et al 2002
Martins	US	North America	Stream	Freshwater	104	342	0.031	Thompson & Townsend 2003
Mill Stream	England/UK	Europe	Stream	Freshwater	87	1654	0.218	Layer et al 2010
Mojave parasitoids	US	North America	Desert, Chaparral	Terrestrial	37	74	0.054	Hawkins & Goeden 1984
Mondego	Portugal	Europe	River Lagoon, Estuarine	Estuary	26	103	0.152	Baeta et al 2011
Monterey Bay	US	North America	Marine Intertidal	Marine	37	79	0.057	Glynn 1965
NorthCalifornia	US	North America	Marine	Marine	80	1446	0.225	Ruzicka 2012
NorthCol	New Zealand	Oceania	Stream	Freshwater	78	241	0.039	Thompson & Townsend 2003
Onca	Brazil	South America	River	Freshwater	40	241	0.150	Angelini et al 2013
Osa scavengers	Costa Rica	Central America	Scavenger food web on toad carrion in tropical wet lowland	Terrestrial	50	131	0.052	Cornaby 1974
Parana	Brazil	South America	River	Freshwater	40	185	0.115	Angelini & Agostinho 2005
Pawnee	US	North America	Prairie	Terrestrial	133	416	0.023	Harris & Paur 1972
Porteirinho	Brazil	South America	Stream	Freshwater	119	310	0.021	Motta & Uieda 2005
Powder	New Zealand	Oceania	Stream	Freshwater	78	268	0.044	Thompson & Townsend 2003
Quick Pond	US	North America	Pond	Freshwater	113	1902	0.148	Preston et al 2012
Saguaro	US	North America	Desert Giant Cactus Forest	Terrestrial	48	138	0.059	Howes 1954
Sarracenia	US/Canada	North America	Pitcher Plant	Aquatic	91	1834	0.221	Baiser et al 2012

Scotia Sea	Chile/Argentina/UK claimed region	Antarctica	Marine	Marine	56	178	0.056	Hopkins et al 1993
Skipwidth	UK	Europe	Pond	Freshwater	37	380	0.277	Warren 1989
St Marks	US	North America	Estuary	Estuary	48	221	0.095	Christian & Luczkovich 1999
St Martin	France/St. Maarten	Caribean/Antilles	Island	Terrestrial	44	218	0.112	Goldwasser & Roughgarden 1993
Stony Stream	New Zealand	Oceania	Stream	Freshwater	112	832	0.066	Townsend et al 1998
Sutton Autumn	New Zealand	Oceania	Stream	Freshwater	80	335	0.052	Thompson & Townsend 2003
Sutton Spring	New Zealand	Oceania	Stream	Freshwater	74	391	0.071	Thompson & Townsend 2003
Sutton Summer	New Zealand	Oceania	Stream	Freshwater	86	423	0.057	Thompson & Townsend 2003
Troy	US	North America	Stream	Freshwater	76	177	0.030	Jarsma et a 1998
UK Grassland	UK	Europe	Grassland	Terrestrial	87	126	0.016	Memmott et al 2000
Venlaw	New Zealand	Oceania	Stream	Freshwater	65	185	0.043	Thompson & Townsend 2003
Vilas	US	North America	Pond	Freshwater	77	958	0.161	Schneider 1997
Wet tropics	Australia	Oceania	River	Freshwater	62	211	0.054	Rayner et al 2010
Afon Hafren	Wales/UK	Europe	Stream	Freshwater	25	135	0.216	Layer et al 2010

2) Temperature estimates when unavailable from GIS layers

Temperature data was unavailable from GIS layers for 6 food webs (Antartica, Chesapeake, Monterey Bay, Stony Stream, Sutton Au, Sutton Sp, Sutton Su). In those cases, I estimated annual average temperatures using data from other publicly available climate databases.

Antarctica food web annual temperature was assumed to be slightly colder than that obtained for the Scotia Sea food web (from package *levitus*) due to their geographic proximity.

Chesapeake Bay food web annual temperature was estimated using temperature data from NOAA's Chesapeake Bay Bridge Station as reported in the Center for Coastal and Physical Coeanography at http://www.ccpo.odu.edu/ccslri/baytemps.pdf (last accessed on Feb 2019).

Monterey Bay food web annual temperature was estimated using NOAA's annual temperature online datasets for the San Francisco Bay-Monterey areas at https://w2.weather.gov/climate/xmacis.php?wfo=mtr (last accessed Feb 2019).

The temperatures for the four New Zealand food webs Stony Stream, Sutton Au, Sutton Sp, Sutton Su where estimated from available annual temperature in nearby town of Clinton, NZ, as reported at https://en.climate-data.org/oceania/new-zealand/otago/clinton-986769/ (last accessed 2019).

3) Statistical methods

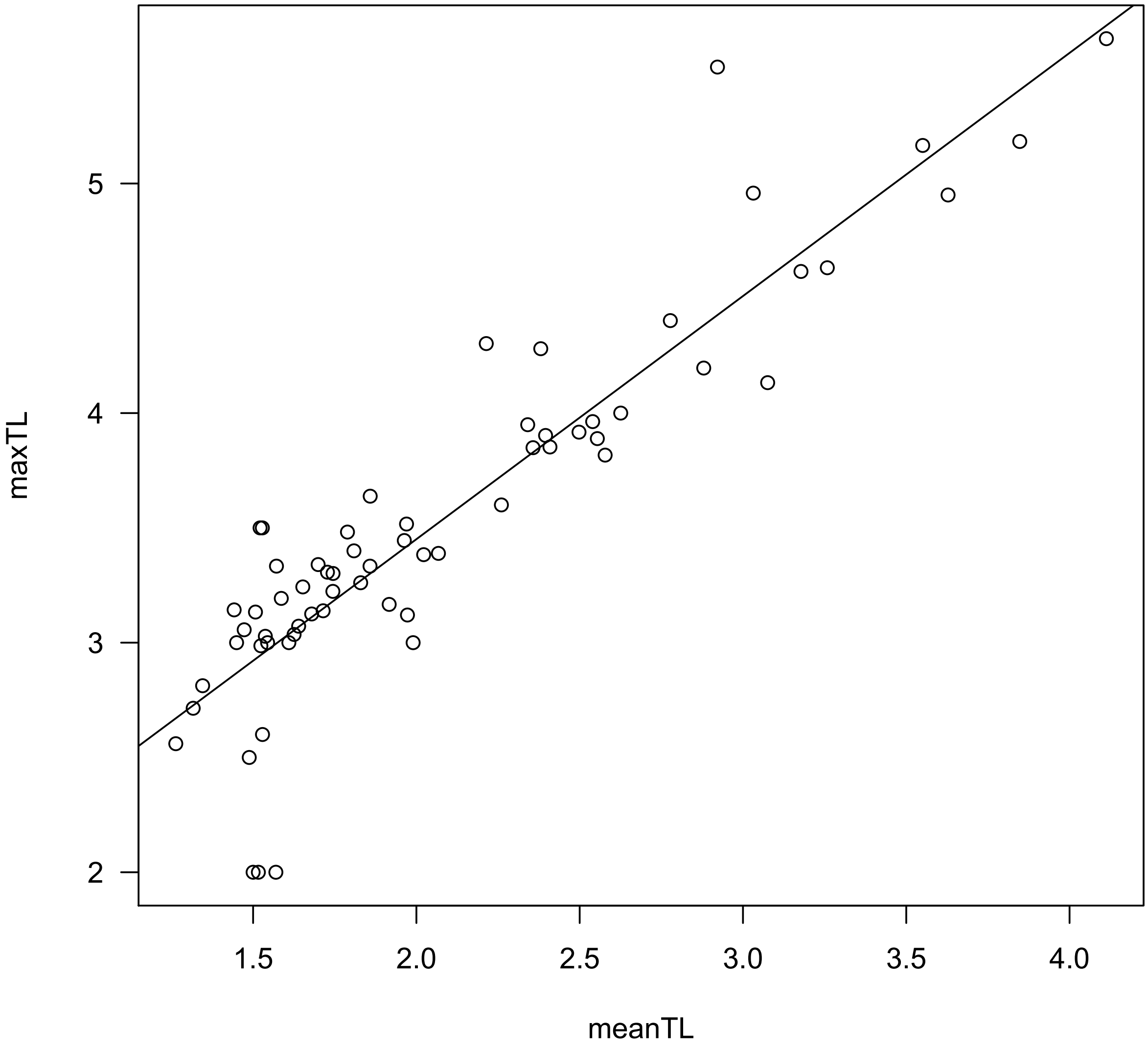

Fig S1: Plot of maxTL against meanTL (estimate=1.05±0.06, pval < 10^{-16}). Notice the slope is basically 1.

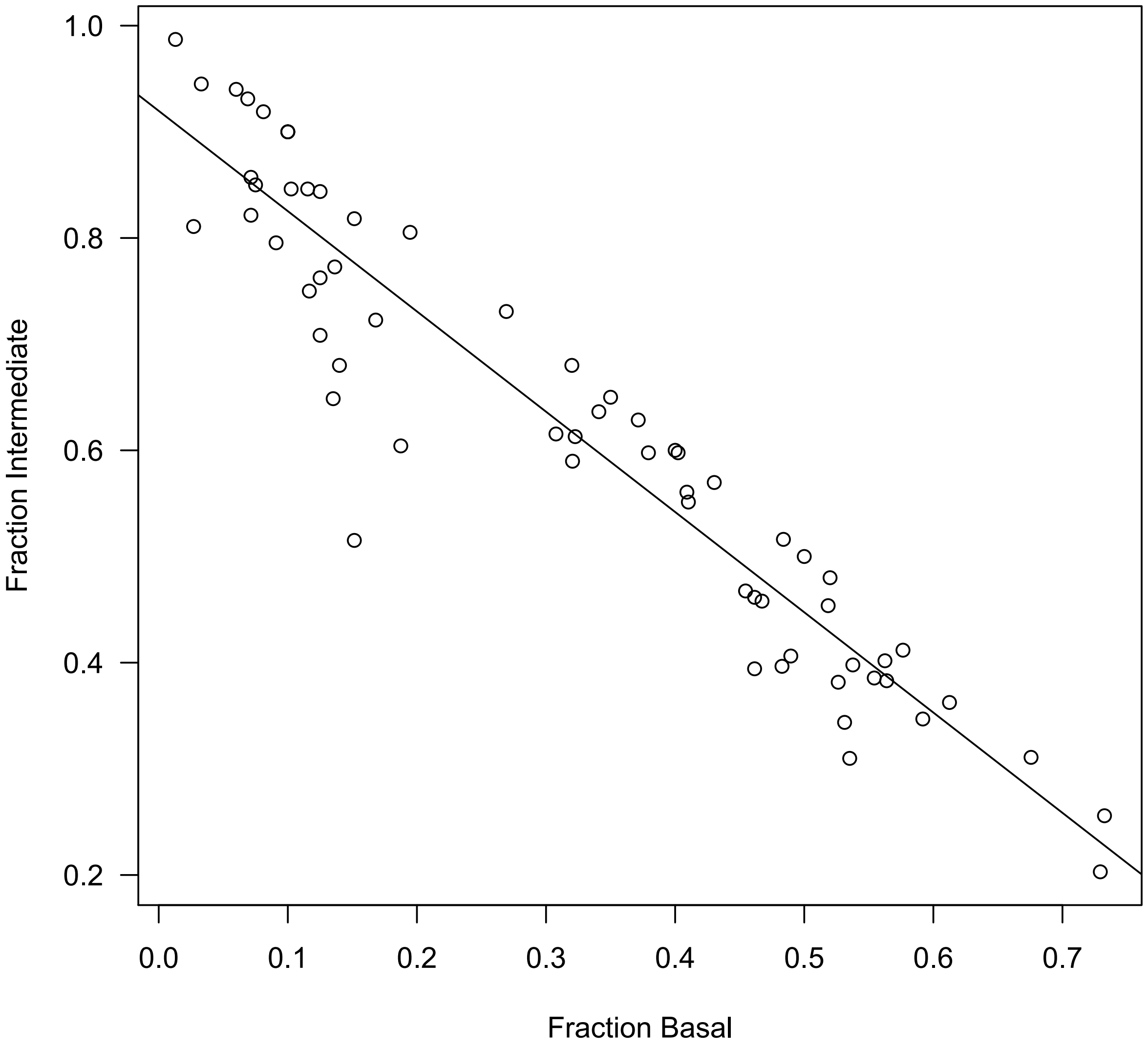

Fig S2: Plot of the fraction of intermediate species against the fraction of basal species (estimate=0.94 ±0.04, pval < 10^{-16}). Notice the slope is basically -1.

Table S2: Latitude was included in absolute value and all variables were standardized (see methods). While ecosystem type is categorical (aquatic or terrestrial), it was converted into a numerical variable (1 or 2). All models were used for aggregate and non-aggregate food webs.

<table>
<tr><th colspan="2">SEM models</th></tr>
<tr><td>Full model (Temperature + Latitude)</td><td>TL ~ Basal + Top + Species + Links + Temp + Lat
C ~ Basal + Top + Species + Links + Temp + Lat
Omnv ~ Basal + Top + Species + Links + Temp + Lat
Links ~ Basal + Top + Species + Temp + Lat + Ecosystem
Top ~ Species + Basal + Temp + Lat + Ecosystem
Basal ~ Species + Temp + Lat + Ecosystem
LS ~ Temp + Lat + Ecosystem
Temp ~ Lat</td></tr>
<tr><td>Temperature only model</td><td>TL ~ Basal + Top + Species + Links + Temp
C ~ Basal + Top + Species + Links + Temp
Omnv ~ Basal + Top + Species + Links + Temp
Links ~ Basal + Top + Species + Temp + Ecosystem
Top ~ Species + Basal + Temp + Ecosystem
Basal ~ Species + Temp + Ecosystem
LS ~ Temp + Ecosystem</td></tr>
<tr><td>Latitude only model</td><td>TL ~ Basal + Top + Species + Links + Lat
C ~ Basal + Top + Species + Links + Lat
Omnv ~ Basal + Top + Species + Links + Lat
Links ~ Basal + Top + Species + Lat + Ecosystem
Top ~ Species + Basal + Lat + Ecosystem
Basal ~ Species + Lat + Ecosystem
LS ~ Lat + Ecosystem</td></tr>
<tr><td>Neither Temp nor Lat model</td><td>TL ~ Basal + Top + Species + Links
C ~ Basal + Top + Species + Links
Omnv ~ Basal + Top + Species + Links
Links ~ Basal + Top + Species + Ecosystem
Top ~ Species + Basal + Ecosystem
Basal ~ Species + Ecosystem
LS ~ Ecosystem</td></tr>
</table>

Calculating direct, indirect and total effects

Once a model is fit and the coefficients for all direct effects are obtained, it is possible to calculate indirect and total effect. Indirect effects would be effects of a variable on another through its direct effects on other intermediate variables, while the total effects would be the sum of its direct and indirect effects on a given variable of interest.

If we assume the existence of three variable, A, B, and C, and if the coefficient for A->B equals 0.5, the one for B->C equals 0.5 and that for A->C equals 0.5, then A directly affects C with a coefficient of 0.5, but it also indirectly affects C through its effect on B with a coefficient equal to 0.5*0.5=0.25. The total effect of A on C would then be 0.5+0.25=0.75.

I used this simple method to quantify all direct and indirect effects of temperature on food webs biotic and network-structural properties (Fig 3 and Figs S2 and S4 of this appendix).

4) Results for aggregated food webs

Table S3: Structural equation model and model descriptors by model (with both latitude and temperature, only latitude or temperature, or neither) and by food web type (aggregated, non-aggregated) ranked by model delta AIC score. Below, I report model chi-square values (χ^2), degrees of freedom (df), p values (here, the larger the better), comparative fit square values (the closer to 1 the better), root mean square errors of approximation (the closer to 0, the better, values above 0.08 are suggestive of a bad fit), standardized root mean square residuals (smaller than 0.08 suggest a good fit), adjusted goodness of fit (can be interpreted as the proportion of explained variance), Aikaike Information Citerion values (AIC) and delta AICs (ΔAIC).

SEM Model	χ^2	df	p-val	Comparative Fit Index	Root Mean Square Error of Approximation	Standardized Root Mean Square Residual	Adjusted Goodness of fit	AIC	ΔAIC
Temperature	2.420	3	0.490	1	0.000	0.016	0.850	363	0
Latitude	2.364	3	0.500	1	0.000	0.016	0.847	375	12
None	2.613	3	0.455	1	0.000	0.018	0.862	382	19
Temp + Lat	2.461	4	0.652	1	0.000	0.018	0.985	423	60

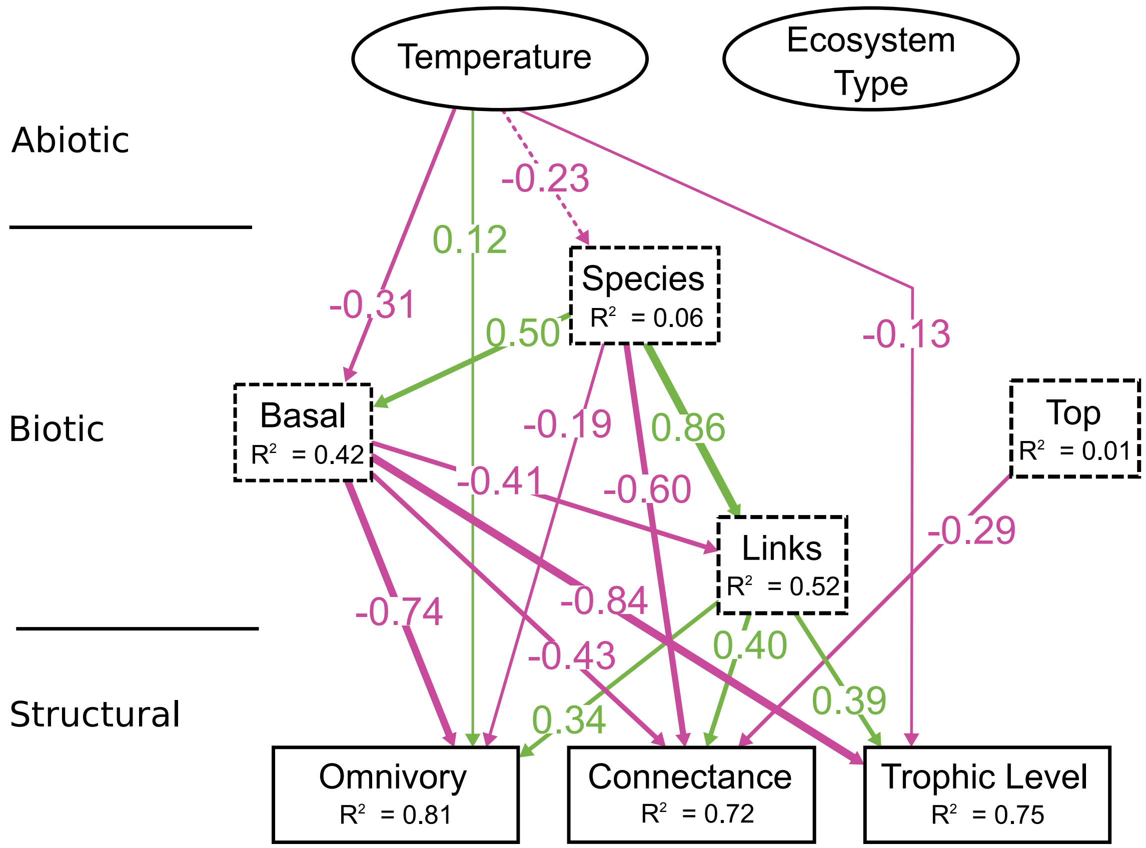

Fig S3: As Fig 2 of the main text but for aggregated food webs. Notice how temperature has now a direct impact on Trophic level but no direct impact on the number of links, while Ecosystem type and the fraction of top species no longer impacts the number of links. Despite these differences, the results are largely unchanged.

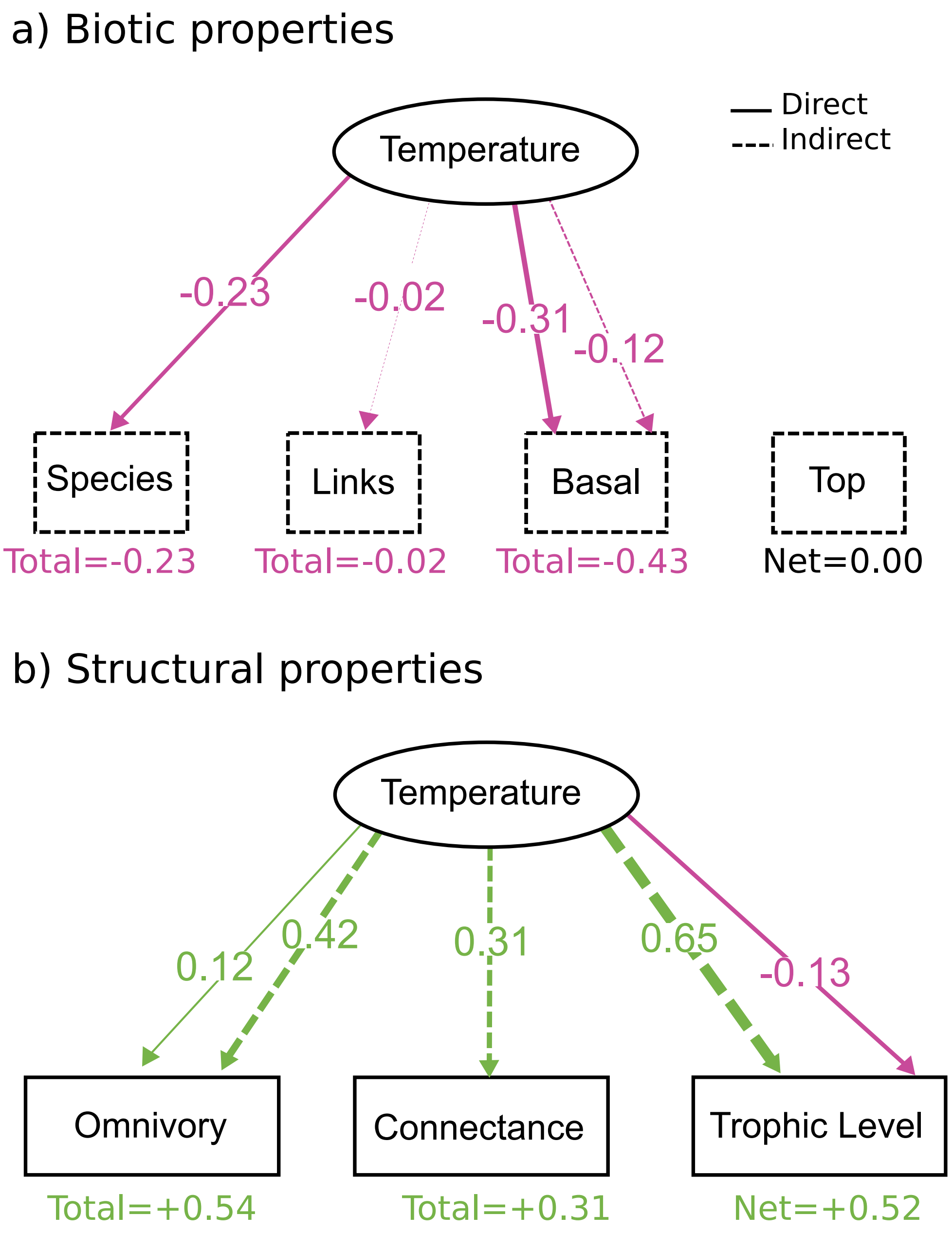

Fig S4: As in Fig 3 of the main text but for aggregated food webs.

5) Results without the FWs for which Temp was unavailable from GIS

Table S4: Structural equation model and model descriptors by model (with both latitude and temperature, only latitude or temperature, or neither) and by food web type (aggregated, non-aggregated) ranked by model delta AIC score. Below, I report model chi-square values (χ^2), degrees of freedom (df), p values (here, the larger the better), comparative fit square values (the closer to 1 the better), root mean square errors of approximation (the closer to 0, the better, values above 0.08 are suggestive of a bad fit), standardized root mean square residuals (smaller than 0.08 suggest a good fit), adjusted goodness of fit (can be interpreted as the proportion of explained variance), Aikaike Information Citerion values (AIC) and delta AICs (ΔAIC).

SEM Model	χ^2	df	p-val	Comparative Fit Index	Root Mean Square Error of Approximation	Standardized Root Mean Square Residual	Adjusted Goodness of fit	AIC	ΔAIC
Temperature	1.572	3	0.666	1	0.000	0.014	0.900	807	0
Latitude	1.476	3	0.688	1	0.000	0.013	0.899	815	8
None	1.564	3	0.668	1	0.000	0.016	0.905	832	25
Temp + Lat	2.162	4	0.706	1	0.000	0.018	0.990	850	43

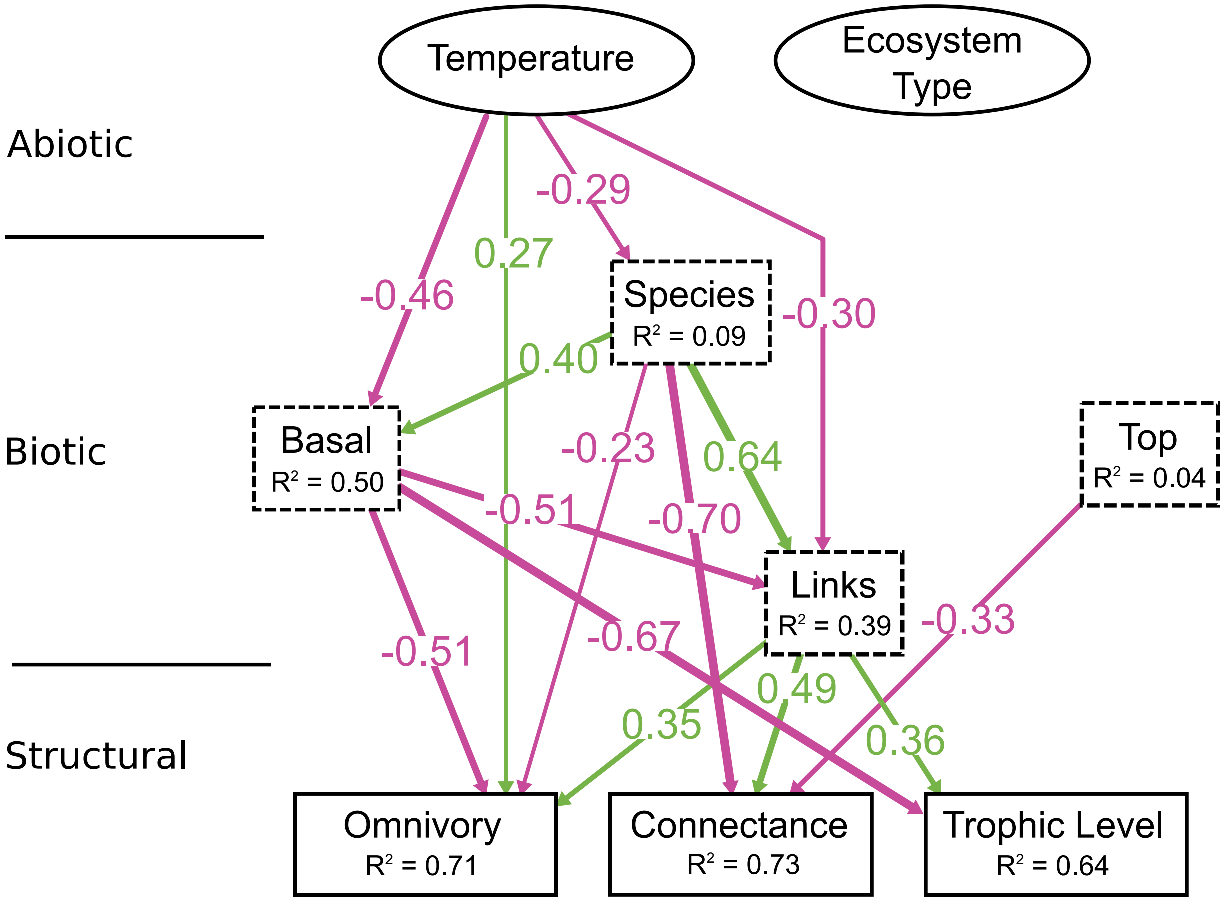

Fig S5: As Fig 2 of the main text but for aggregated food webs. Notice how Ecosystem type and the fraction of top species no longer impact the number of links. Also, the fraction of basal species no longer impacts connectance. Despite these differences, the results are largely unchanged.

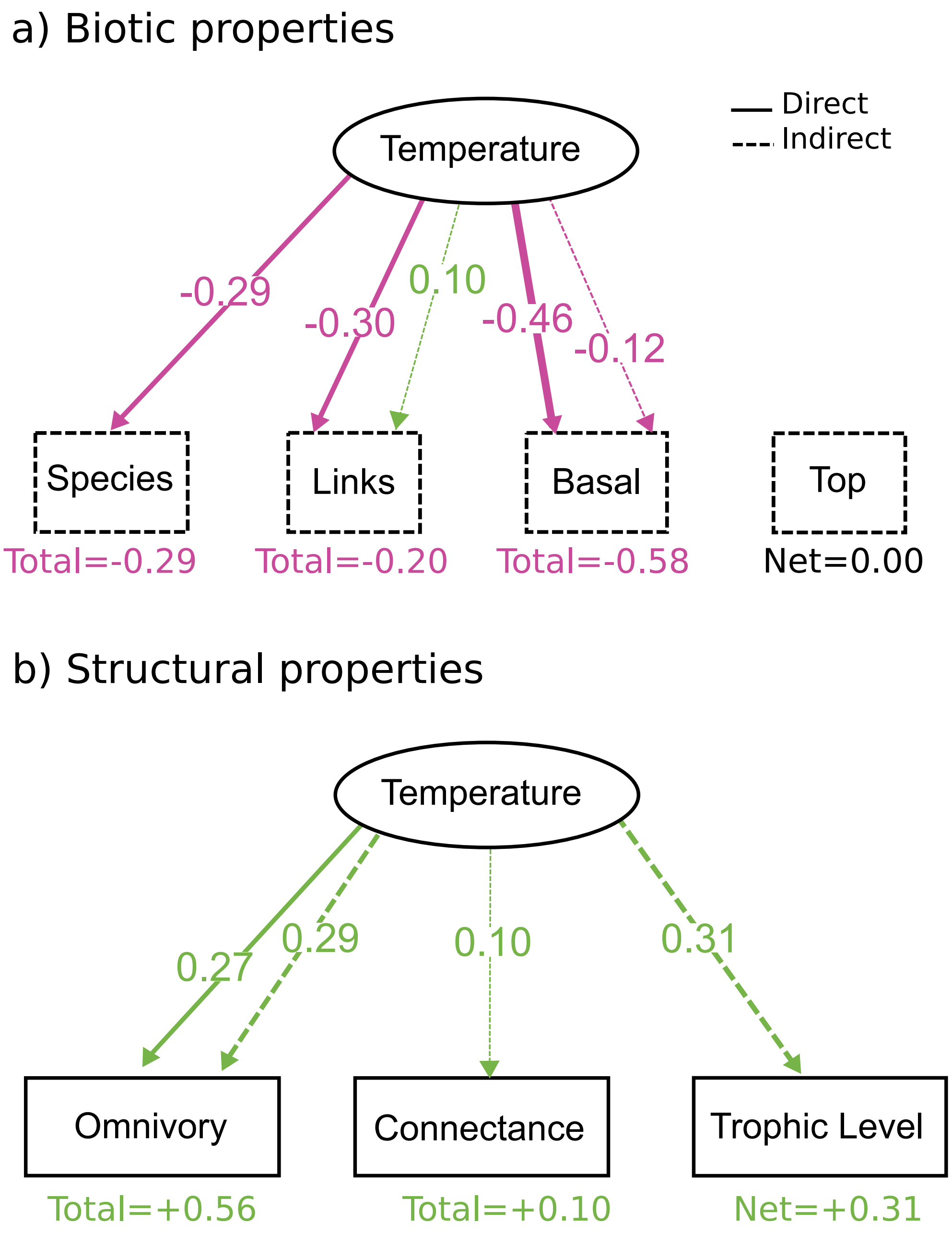

Fig S6: As in Fig 3 of the main text but for aggregated food webs.
